# Unraveling the multifaceted benefits of physical exercise: a comprehensive review of body composition, metabolic regulation, and systemic health

**DOI:** 10.3389/fnut.2025.1691690

**Published:** 2026-01-05

**Authors:** Shengxuan Zhang, Inmaculada Xu Lou, Sammit Vishram Deshpande, Lei Sun, Ali Hamza, Kamran Ali, Qilan Chen

**Affiliations:** 1 Department of Cardiology, Hangzhou Hospital of Traditional Chinese Medicine, Hangzhou, Zhejiang, China; 2 Deshpande Clinic, Talegaon Dabhade, Pune, Maharashtra, India; 3 Zhejiang Chinese Medical University, Hangzhou, Zhejiang, China; 4 Department of Gastroenterology, Hangzhou Hospital of Traditional Chinese Medicine, Hangzhou, Zhejiang, China; 5 Department of Zoology Wildlife and Fisheries, University of Agriculture, Faisalabad, Pakistan; 6 Department of Surgery, The Fourth Affiliated Hospital of School of Medicine, and International School of Medicine, International Institutes of Medicine, Zhejiang University, Yiwu, Zhejiang, China

**Keywords:** bariatric surgery, diet control, lifestyle modifications, high intensity interval training, obesity

## Abstract

Obesity, a significant comorbidity for various cardiovascular and respiratory diseases, demands individualized and effective management strategies. Without appropriate intervention, obesity can severely compromise an individual’s health. Achieving weight management is feasible through the application of evidence-based knowledge and sustained commitment. Incorporating diverse forms of physical exercise—such as High-Intensity Interval Training (HIIT) and Moderate-Intensity Continuous Training (MICT)—in combination with tailored dietary habits can optimize outcomes for affected individuals. In fact, dietary regulation remains the cornerstone of any weight management program, especially among adolescents and adults facing modern lifestyle challenges. When paired with an exercise regimen aimed at reducing adipose tissue, this combined approach effectively facilitates weight control. Moreover, obesity is characterized by chronic low-grade systemic inflammation and is closely linked to numerous metabolic disorders, including the highly prevalent Diabetes Mellitus. This connection is largely attributable to obesity’s profound impact on hormonal regulation, particularly in the development of insulin resistance. For individuals who are unable to manage their weight through conventional means, bariatric surgery may be considered in advanced cases. However, post-surgical care—comprising proper dietary management and regular physical exercise—is essential for achieving and maintaining favorable outcomes. In this literature review, recent articles from the past 5 years examining the benefits of physical exercise on obesity were analyzed. The findings reveal that different modalities of physical exercise influence weight loss, adipose tissue reduction, body composition, metabolism, physical capacity, cardiorespiratory function, insulin regulation, inflammation, psychological adaptations, hormonal balance, gut microbiota, as well as factors related to pregnancy and aging. Notably, the benefits derived from physical exercise vary according to the specific type of activity performed. Consequently, when prescribing an exercise regimen, it is crucial to align the program with the individual’s specific therapeutic objectives.

## Highlights

- **What is already known on this topic**: Obesity is a complex global health issue that significantly increases the risks of debilitating diseases. While exercise and nutrition modifications remain integral to mitigating negative health impacts, finding scalable personalized solutions has proved challenging.
- **What this study adds**: Here we synthesize recent evidence on how targeted exercise interventions influence metabolic regulation, inflammation, and hormonal signaling, highlighting tailoring approaches to individual health profiles. From brief high-intensity sessions to moderate duration activities, specific modalities demonstrate potential when matched to circumstances.
- **How this study might affect research, practice, or policy**: These findings underscore the value of personalized regimens that leverage clinical insights and individual characteristics. With care providers and policymakers better informed on evidence-based strategies, more holistic yet targeted efforts can be developed and scaled to improve outcomes in obesity prevention, management, and community wellbeing.

## Introduction

Obesity remains a significant global public health concern, serving as a precursor to numerous chronic diseases (1). To address this issue, various strategies have been implemented, with physical exercise being one of the most promising (2). Despite its benefits, adherence to exercise programs is generally low, particularly among females, older individuals, and those with lower educational levels (3). Lifestyle modifications that include regular physical activity not only offer substantial clinical benefits for metabolic syndrome but may also yield cost savings by reducing the need for medications (4). Current guidelines recommend at least 300 min of moderate-intensity activity per week for weight loss, with evidence suggesting that a combination of high-intensity aerobic and resistance training produces optimal results (5).

**Figure 1:**
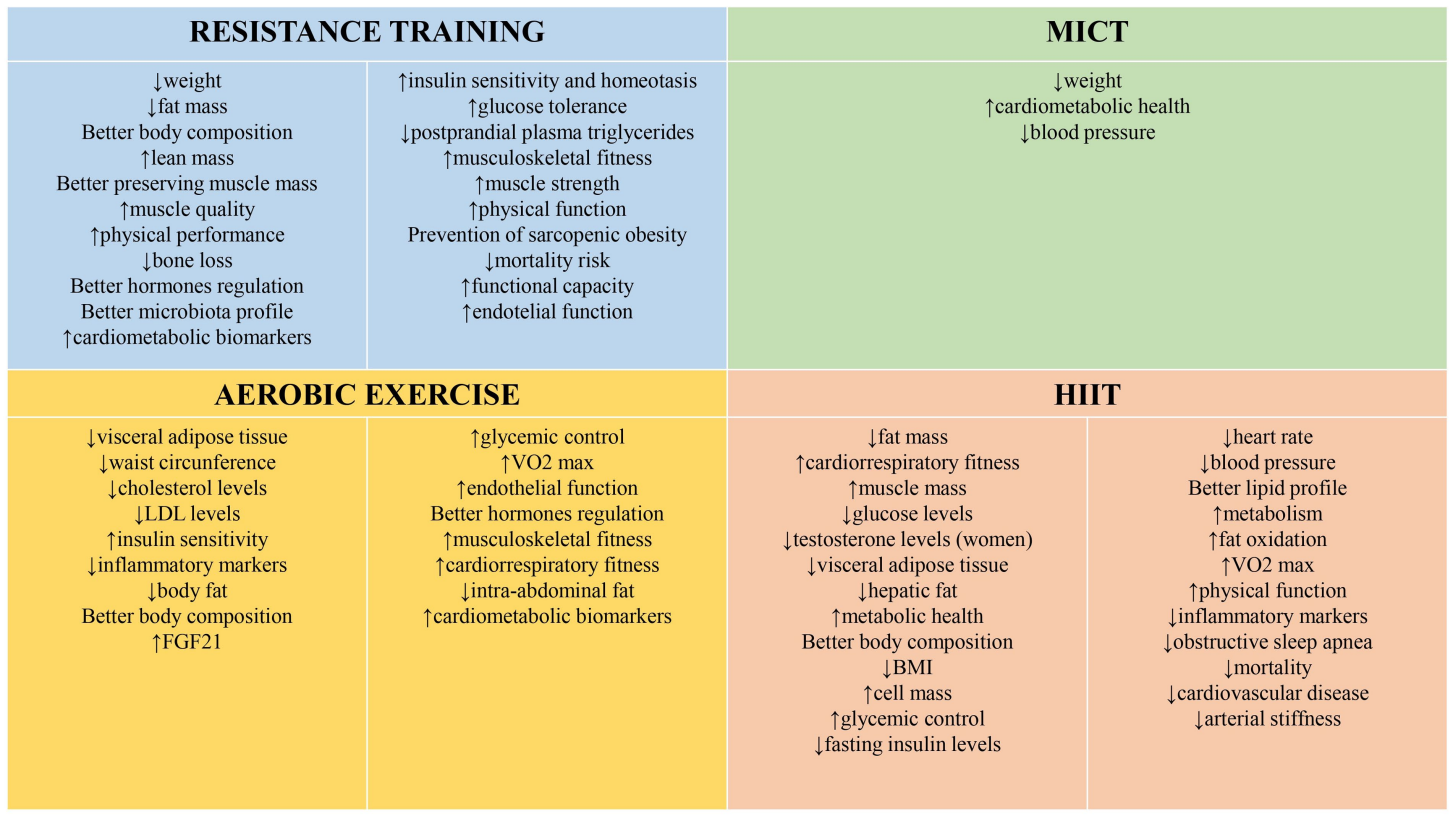
Multifaceted effects of physical exercise.

For this updated review, we conducted a literature search using the PubMed database. Inclusion criteria were studies published between 2019 and 2024 focusing on the effects of physical exercise on overall health and obesity, conducted in human populations, and categorized as randomized controlled trials (RCTs), systematic reviews, or meta-analyses. Figure 1 summarizes the benefits of engaging in physical exercise.

## Weight loss through caloric restriction

Caloric restriction, while capable of producing initial weight loss, often results in a reduction of lean mass and strength—particularly among older adults (>65 years)—making it an insufficient stand-alone strategy (6). Multiple studies have examined the role of exercise in enhancing weight loss and preventing weight regain. For example, Berge et al. (2021) compared moderate-intensity continuous training (MICT) with a combination of MICT and high-intensity interval training (HIIT) over 24 weeks in obese women. The combined MICT/HIIT group lost an additional 3 kg compared to the MICT-only group, despite similar exercise energy expenditures (7). Similarly, circuit training effectively reduced BMI in obese individuals, although similar benefits were not observed in those with normal weight (8).

Following bariatric surgery, weight regain is a common challenge. Postoperative exercise can mitigate this by reducing fat mass, blood glucose, and cholesterol levels (9). While early resistance training may not significantly improve long-term muscle strength, increasing moderate-intensity physical activity is essential for weight maintenance (10). A strong association exists between engaging in moderate to vigorous physical activity (at least 150 min per week) and the prevention of weight gain, though more evidence is needed to evaluate the efficacy of light-intensity activity (11). Moreover, interventions that integrate exercise with dietary changes—such as combining strength/resistance training with a personalized hypocaloric diet—have demonstrated superior effectiveness in managing obesity in adults (12).

Among pregnant women with obesity, physical activity has been shown to limit excessive gestational weight gain (13). Factors such as being male, older in age, cardiometabolic comorbidities, and dietary fat intake also influence weight loss success (14). Short-term programs that combine exercise with nutritional interventions have proven effective for obese adults, and post-weight loss strategies emphasizing physical training with adequate protein, calcium, and vitamin D intake help prevent weight regain (15, 16).

Although exercise interventions favorably impact weight, BMI, and visceral adipose tissue (VAT), exercise alone often produces less weight loss than anticipated—possibly due to compensatory increases in energy intake (17, 18). In some cases, incorporating severe energy restriction into dietary interventions enhances the effectiveness of exercise in reducing body weight (19). Combined interventions (diet plus physical activity) have shown similar weight loss outcomes with reduced weight regain compared to traditional aerobic exercise alone (20). Additionally, while both morning and evening exercise sessions can promote weight loss, improvements in VO₂ peak are achieved regardless of exercise timing, with no clear optimal time identified (21).

## Effects on adipose tissue

HIIT has consistently been shown to induce fat loss and improve cardiorespiratory fitness in obese women. For instance, HIIT combined with caffeine supplementation reduced body fat percentage and improved metabolic parameters in women with over 40% body fat (22). Similar benefits were observed in older obese individuals, with progressive HIIT reducing both fat mass and VAT while increasing lean mass (23). Likewise, resistance training paired with a hypocaloric diet effectively reduced overall body weight and fat mass (24). Combining moderate-intensity aerobic exercise with resistance training and HIIT appears to be more effective in reducing VAT than any single modality (25), and regular aerobic exercise alone has been associated with significant reductions in waist circumference (26).

Interventions that merge exercise with dietary modifications generally yield greater improvements in lipid profiles and VAT reduction than either approach alone (27). Although time-restricted eating reduces body weight, it may adversely affect blood lipid levels and lean tissue mass (28). Other strategies, such as combining taurine supplementation with exercise has been shown to boost lipid oxidation and mitochondrial function, potentially inducing ‘browning’ of subcutaneous adipose tissue in obese women (29). In addition, employing blood flow restriction (BFR) during HIIT sessions can further enhance VAT reduction and improve glucose metabolism, likely due to elevated lipolytic hormone levels (30).

Physical exercise and low-calorie diets create an energy deficit that influences both visceral and subcutaneous fat. For every 1 kg of total fat lost, there is an approximate reduction of 10 cm^2^ in abdominal subcutaneous fat (31). Even walking, independent of other interventions, has been shown to improve lipid profiles by reducing total cholesterol, LDL, and triglycerides, while increasing HDL (32). Furthermore, HIIT has proven effective in reducing hepatic fat and enhancing metabolic health in obese adults, even without significant weight loss (33), although VAT reduction via HIIT at 90% VO₂peak may be insensitive to changes in training intensity (34). Vigorous physical activity uniquely reduces cardiac adipose tissue volume (35), and when combined with caloric restriction, exercise exhibits a dose–response effect on VAT reduction—unlike caloric restriction alone (36). Moderate-intensity aerobic exercise has been shown to effectively reduce total cholesterol and LDL, whereas high-intensity exercise may significantly lower adiponectin levels in middle-aged women with obesity (37).

## Modifications in body composition

Recent studies indicate that resistance and strength training have positive effects on body composition, with combined exercise strategies outperforming isolated interventions (38). For example, Chiang et al. (2019) compared a 12,000-steps-per-day regimen with a combination of walking and moderate-intensity training over 8 weeks in college students with obesity; the combined approach was more effective in improving body composition and metabolic syndrome parameters (39). Stair climbing exercises, performed three times weekly over 12 weeks, have similarly improved insulin sensitivity and reduced inflammatory markers in obese women (40). Additionally, combining physical exercise with intermittent fasting has shown enhanced benefits on body composition and cardiometabolic health, although the effects on cardiometabolic markers may not differ significantly from exercise alone (41).

HIIT has also been highlighted for its significant impact on body composition. A 4-week HIIT program among obese university students resulted in notable improvements, including reduced BMI, decreased body fat percentage, and increased lean and skeletal muscle mass (42). High-intensity physical activity is generally more effective than moderate-intensity exercise in reducing body fat and increasing muscle mass, particularly when combined with a hypocaloric diet (43, 44). Studies suggest that both low- and high-intensity aerobic and anaerobic training reduce body fat while increasing lean body mass and VO₂max (45). Resistance training, especially when combined with caloric restriction, is particularly effective in reducing fat mass and preserving muscle mass—benefits that are more pronounced in younger adults (46–48). In a study conducted in men, combined strength and resistance training was shown to confer long-term benefits in maintaining lower body fat levels (49).

For individuals undergoing bariatric surgery, combining diet with resistance training can help preserve muscle mass and improve muscle quality (50). In sarcopenic obesity, resistance training is crucial for enhancing physical performance (51). When paired with time-restricted eating or supplementation (e.g., astaxanthin), physical training can further reduce fat mass and improve metabolic profiles (52, 53). Additionally, integrating HIIT with intermittent fasting may lead to superior reductions in body mass compared to HIIT alone (54). Post-bariatric surgery, physical training can restore muscle strength and improve muscle structure and function, closely approximating the condition of lean, healthy individuals (55). In a study conducted in women, exercise was also shown to play a key role in mitigating bone loss after bariatric surgery by suppressing bone turnover and reducing sclerostin levels, which is critical for preserving bone mineral density (56). Furthermore, resistance training combined with omega-3 polyunsaturated fatty acid supplementation has shown benefits on muscle function, body composition, and cardiometabolic profiles, particularly in postmenopausal women (57).

## Modifications in metabolic regulation

Obesity is closely linked with metabolic disorders such as type 2 diabetes and cardiovascular diseases. Elevated chemerin levels, which contribute to these disorders, are significantly reduced following physical training—a change that correlates with decreases in body fat percentage (58). Exercise also influences Fibroblast Growth Factor 21 (FGF21), with acute bouts increasing circulating levels and chronic programs enhancing FGF receptor expression in key tissues (59). In obese adolescents, a 6-month supervised exercise program has been shown to reduce low-grade systemic inflammation, which may help prevent metabolic diseases (60). Moreover, physical training reduces postprandial glucose and insulinemia in adults with obesity, irrespective of age or baseline glucose levels (61). HIIT and resistance exercises have similarly demonstrated improvements in postprandial lipid oxidation, glucose metabolism, and insulin sensitivity (62–64). Interval training, in particular, appears to produce superior improvements in peak VO₂ and fat oxidation compared to continuous exercise (65, 66). In postmenopausal women and older obese adults, regular physical activity significantly improves multiple risk factors for metabolic syndrome and reduces overall cardiometabolic risk (67, 68). Even short bouts of walking before peak postprandial glucose levels can lower glucose, insulin, and C-peptide concentrations (69).

Extended exercise programs (≥12 weeks) may slightly increase fasting hunger, a factor that should be considered when designing interventions to prevent compensatory energy intake (70). Liver-expressed antimicrobial peptide-2 (LEAP-2) has emerged as a key regulator of energy balance and glucose metabolism. Initially identified in 2003 as an endogenous antagonist of the acylated ghrelin receptor, LEAP-2 inhibits ghrelin binding to its growth hormone secretagogue receptor (GHSR). Subsequent studies have demonstrated that exogenous LEAP-2 infusion acts as a satiety signal, reducing food intake and lowering plasma glucose levels in humans (71). Collectively, these findings suggest that LEAP-2 plays a pivotal role in appetite regulation and may represent a potential therapeutic target for obesity. Furthermore, alterations in LEAP-2 levels following interventions combining caloric restriction and intermittent exercise have been associated with decreased food cravings and improved aerobic capacity in women with obesity (72).

## Improvements in physical functionality

Hybrid and multicomponent interval training programs have been shown to improve musculoskeletal fitness in overweight adults in a dose-dependent manner (73). Resistance training significantly enhances muscle strength and, to a moderate degree, overall physical function, although concurrent caloric restriction might blunt some functional adaptations (74). Obesity adversely affects gait parameters—increasing the risk of falls—while combining exercise with caloric restriction yields improvements in walking speed, particularly in older adults (75, 76). Exercise interventions effectively reduce pain and improve physical function in individuals with obesity-related knee osteoarthritis, thereby enhancing overall quality of life (77). These benefits occur through multiple mechanisms, including weight reduction, increased muscle strength and joint stability, decreased systemic and local inflammation, and improved mobility. Furthermore, regular physical activity promotes gains in lean muscle mass, muscle power, physical function, and balance. Weight loss achieved through exercise also contributes to a sustained reduction in joint pain (77, 78).

## Effects on the cardiovascular and respiratory systems

Low-volume HIIT has demonstrated significant improvements in cardiorespiratory capacity, even though changes in total body fat or lean mass may not differ significantly from those observed in non-exercising or MICT groups (79). HIIT typically produces moderate reductions in both central and peripheral blood pressure, whereas MICT tends to improve only peripheral measures (80). Short intervals (e.g., 30-s HIIT bouts) have been shown to improve systolic blood pressure, maximal heart rate, and inflammatory markers such as CRP in obese youth (81). Low-volume HIIT protocols can also induce significant improvements in VO₂max and overall well-being over a 12-week period (82).

In adults, aerobic exercise appears to be more effective at enhancing cardiorespiratory fitness and body weight regulation, while low-load resistance training more effectively reduces body adiposity (83). Combining exercise with a low-carbohydrate diet improves cardiometabolic profiles, although it may result in greater muscle mass loss (84). Conversely, a calorie-restricted diet combined with interval training has been associated with improvements in arterial stiffness, peak VO₂, and HDL levels (85). Regular physical activity also prevents gestational hypertension and supports cardiovascular health in overweight pregnant women (86). Improvements in endothelial function and flow-mediated dilation following aerobic exercise further contribute to reducing coronary artery disease risk in obese individuals (87). For postmenopausal women, moderate walking programs have been shown to reduce inflammatory markers and adiposity, thereby contributing to cardiovascular disease prevention (88). Additionally, patients with severe obesity exhibit notable improvements in cardiorespiratory fitness following exercise interventions post-bariatric surgery (89). Genetic factors, such as variants of the β2-adrenergic receptor (ADRB2), may interact with aerobic training to enhance cardiac autonomic function and other health outcomes in obesity (90). Finally, HIIT has been shown to reduce cardiovascular risk factors—such as blood pressure, arterial stiffness, and lipid abnormalities—in hypertensive obese women. Moreover, performing HIIT in hypoxic environments (e.g., simulated altitude) may offer additional improvements, potentially by enhancing vascular adaptation, endothelial function, and oxygen utilization efficiency (91, 92).

## Regulation of insulin levels

Obesity is strongly associated with insulin resistance and the development of type 2 diabetes. Studies consistently demonstrate that combined physical exercise and dietary interventions are more effective at improving insulin resistance and fasting glucose levels than either strategy alone (93). Regular, rather than sporadic, exercise improves insulin sensitivity (94). In obese men with prediabetes, aerobic training before a mixed meal test has been shown to reduce postprandial glucose appearance and plasma insulin levels (95). Similarly, concurrent resistance and aerobic training, particularly when combined with caloric restriction, significantly enhances insulin sensitivity in premenopausal obese women (96). HIIT has also been identified as an effective strategy for reducing fasting insulin levels and HOMA-IR in obese men following a 12-week program (97).

## Effect on obesity-associated inflammation

Obesity is characterized by chronic low-grade systemic inflammation. Various exercise modalities—including HIIT, aerobic, and combined regimens—have been shown to reduce inflammatory biomarkers such as TLR-4, IL-1β, IL-18, CRP, IL-6, leptin, and TNF-*α*. These anti-inflammatory effects are often correlated with improvements in aerobic capacity and are particularly pronounced in populations with elevated BMI (98–100).

## Effect on hormone regulation

Physical exercise modulates key hormones involved in hunger and energy homeostasis. Different exercise modalities affect ghrelin levels in distinct ways; for example, circuit and interval resistance training appear to better regulate ghrelin and other obesity-related hormones compared to traditional resistance training (101). Combining a hypocaloric diet with interval exercise has been shown to suppress acyl ghrelin and reduce hunger perceptions in obese women (102). In addition, continuous exercise may enhance postprandial responses of hormones such as GLP-1 and PYY, while dietary interventions more effectively modulate leptin and adiponectin levels compared to exercise alone (103). Exercise also influences sex hormone levels; in adolescents, regular exercise has been associated with increased adiponectin, decreased resistin, and a reduced rate of pubertal progression—effects that are reversed upon detraining (104).

## Effects on the microbiota

Emerging evidence suggests that physical exercise positively modulates gut microbiota diversity, an important factor in metabolic health. Exercise increases the abundance of beneficial bacteria such as *Bifidobacteriaceae*, *Bacteroides*, and *Akkermansia*, while reducing *Proteobacteria*—organisms linked to obesity and type 2 diabetes (105). Resistance training, for example, has been associated with an increased abundance of *Roseburia*, a key producer of short-chain fatty acids, which are critical for gut health (106).

## Conclusion

Collectively, recent research underscores that physical exercise—whether as a stand-alone intervention or in combination with dietary modifications—produces robust benefits for weight management, body composition, metabolic regulation, cardiovascular health, and inflammation in obese individuals. Future studies should continue to refine exercise prescriptions tailored to different populations and examine the long-term sustainability of these interventions.
